# Efficacy of visualized reamer foraminoplasty in transforaminal endoscopic lumbar discectomy: a retrospective controlled study

**DOI:** 10.3389/fsurg.2026.1806067

**Published:** 2026-04-22

**Authors:** Honglin Liu, Yuxiang Hu, Yang Zhan, Zhixin Kang, Zhuoxuan Zhang, Chengyu Huang, Ningjing Zeng, Yang Xiao, Juntao Ma, Zibo Gao, Hao Liu, Guoyi Su, Yongpeng Lin, Zhirong Fan, Dingkun Lin, Yihao Liang, Yongjin Li

**Affiliations:** 1 The Second Clinical School of Guangzhou University of Chinese Medicine, Guangzhou, China; 2 Gansu University of Chinese Medicine, Lanzhou, China; 3 Guangdong Provincial Hospital of Chinese Medicine, Guangzhou, China; 4 The Second Affiliated Hospital of Guangxi University of Chinese Medicine, Nanning, China

**Keywords:** foraminoplasty, lumbar disc herniation, minimally invasive spine surgery, transforaminal endoscopic lumbar discectomy, visualized reamer

## Abstract

**Objectives:**

The purpose of this study was to retrospectively compare the efficacy and safety of visualized reamer foraminoplasty vs. the traditional TESSYS technique in transforaminal endoscopic lumbar discectomy (TELD) for lumbar disc herniation (LDH).

**Methods:**

In this retrospective study, 140 LDH patients were assigned to the visualized reamer group (*n* = 70) or the TESSYS group (*n* = 70). Perioperative parameters (operative time, fluoroscopy frequency), clinical outcomes [Visual Analog Score (VAS) for back/leg pain, Oswestry Disability Index (ODI)] were assessed preoperatively and at 1 day, 1, 3, 6, and 12 months postoperatively. Peri-operative complications were recorded. Subgroup analyses were performed.

**Results:**

The visualized reamer group demonstrated significant advantages in operative time (66.34 ± 7.65 vs. 76.06 ± 15.89 min, *P* < 0.05) and intraoperative fluoroscopy frequency (6.10 ± 0.90 vs. 12.06 ± 0.92, *P* < 0.05). Both groups showed significant clinical improvement at all timepoints (*P* < 0.05). The visualized reamer group demonstrated superior early postoperative leg pain relief, evidenced by significantly lower VAS leg scores at 1 day (mean difference: −0.64 points) and 1 month (mean difference: −0.43 points) compared to the TESSYS group (both *P* < 0.05). Mid-term clinical outcomes were comparable between the two groups. Complication analysis revealed a significantly lower incidence of postoperative lower limb dysesthesia in the visualized reamer group (0% vs. 5.71%, *P* < 0.05), while recurrence rates showed no significant difference (1.43% vs. 2.86%, *P* > 0.05). Subgroup analyses confirmed consistent treatment benefits across age, BMI, and sex (*P* > 0.05).

**Conclusion:**

The visualized reamer technique significantly enhances perioperative efficiency and early pain control in TELD, with comparable mid-term efficacy to the TESSYS technique. Its benefits are consistent across diverse patient populations, supporting its broad applicability.

## Introduction

Lumbar disc herniation (LDH) is a prevalent degenerative condition of the lumbar spine (1) and a leading cause of disabling low back and leg pain. The condition typically manifests as low back pain, radicular leg pain, and neurological deficits, which can severely impair function and quality of life (2). Furthermore, the global incidence of LDH has been rising in recent years, with a notable increase among younger populations, thereby imposing significant medical and economic burdens (3, 4). Consequently, significant advancements have been made in minimally invasive spine surgery techniques. Among these, transforaminal endoscopic lumbar discectomy (TELD) has emerged as a mainstream minimally invasive procedure for LDH owing to its minimal trauma, rapid recovery, and well-established efficacy (5, 6). A critical step in TELD is foraminoplasty, which aims to enlarge the intervertebral foramen to establish a safe working channel and achieve neural decompression; its precision directly influences surgical visibility, the adequacy of decompression, and overall safety (7). In transforaminal approaches, the YESS technique proposed by YEUNG et al. (8) established the “outside-inside” concept, thereby laying the foundation for minimally invasive spine surgery for LDH. The TESSYS technique, developed by Thomas Hoogland, represents a well-established procedure (9), however, it relies on intraoperative fluoroscopy to guide for foraminoplasty. Since this approach does not allow real-time visualization of surgical instruments relative to critical tissues like nerves and dura, it is associated with a steep learning curve and potential nerve injury risks (10, 11). To address these limitations, visualized foraminoplasty techniques based on TESSYS approach have been developed. Central to this advancement is the visualized reamer, which features an outer protective working sleeve with a built-in endoscope. It is noteworthy that, similar to traditional reamers, the visualized reamer is also a reusable instrument. this design enables surgeons to directly visualize bony structures, adjacent soft tissues, and neurovascular elements in high definition, facilitating precise and controlled bone resection. As a results, fluoroscopy is required only for the initial instrument channel placement, while the entire critical foraminoplasty procedure is performed under continuous endoscopic visualization. This evolution represents a shift from a “fluoroscopy-guided” to a “direct vision-guided” technique.

## Materials and methods

### Study design and participants

This single-center retrospective cohort study was approved by the Ethics Committee of Guangdong Provincial Hospital of Chinese Medicine (Approval No: ZE2025-151-01). We retrospectively analyzed the clinical data from patients who underwent single-level TELD for LDH at the Minimally Invasive Spine Surgery Center between January 2022 and June 2024. All surgeries from January 2022 to August 2023 utilized the TESSYS foraminoplasty technique, while those from August 2023 to June 2024 utilized the visualized reamer technique. To control for surgical skill variation, all procedures were performed by the same surgeon with over 20 years of experience in minimally invasive spine surgery.

The inclusion criteria were as follows: (1) patients with clear unilateral radicular symptoms caused by LDH; (2) single-level LDH confirmed by MRI/CT, consistent with the symptomatic level; (3) failure of ≥3 months of standardized conservative treatment; (4) no previous history of lumbar surgery; (5) availability of complete follow-up data for at least 12 months. (6) For patients with L5-S1 disc herniation, those who met the following criteria on preoperative imaging were included and underwent TELD: iliac crest height below the level of the L4/5 intervertebral space, and herniation type being central, paracentral, or shoulder-type with mild caudal migration (12). Patients were excluded for the following reasons: (1) concomitant lumbar fracture, infection, tumor, or tuberculosis; (2) lumbar spondylolisthesis or instability requiring fusion surgery; (3) far-lateral disc herniations (extraforaminal); (4) multi-level LDH; (5) incomplete or missing follow-up data.

### Data collection

Data for this study were retrospectively extracted from the institutional electronic medical record system. The collected parameters included: (1) baseline characteristics (age, sex, body mass index, operative level); (2) perioperative parameters (operative time, frequency of intraoperative fluoroscopy, intraoperative blood loss, total hospital stay, postoperative length of stay); (3) efficacy outcomes, including Visual Analog Scale (VAS) scores for back and leg pain and Oswestry Disability Index (ODI) scores, which were assessed preoperatively and at 1 day, 1 month, 3 months, 6 months, and 1 year postoperatively; patient satisfaction was evaluated using the modified MacNab criteria (13) at the final 1-year follow-up. The postoperative VAS and ODI assessments, as well as the modified MacNab evaluation, were conducted by an independent clinical researcher who was not involved in the surgery and was unaware of the group assignments.; (4) safety outcomes, which involved monitoring for postoperative complications throughout the study period, including nerve injury, lower limb dysesthesia, cerebrospinal fluid leak, symptomatic hematoma, and symptom recurrence.

### Statistical analysis

All statistical analyses were performed using SPSS Statistics version 27.0. Continuous variables are presented as mean ± standard deviation, while categorical variables are expressed as numbers (percentages). Intergroup comparisons were conducted using independent samples t-tests (continuous variables) or chi-square/Fisher's exact tests (categorical variables). Intragroup comparisons across different time points were analyzed using paired t-tests. A two-sided *P*-value < 0.05 was considered statistically significant.

To assess the consistency of the treatment effect, a prespecified subgroup analysis was performed based on age (≤60 years vs. >60 years), body mass index (BMI, ≤25 kg/m^2^ vs. >25 kg/m^2^), and sex (male vs. female). The outcomes of interest for this analysis were the 12-month postoperative scores and the change scores (Δ) from baseline for the key clinical measures. Heterogeneity of the treatment effect across subgroups was evaluated by introducing an interaction term(treatment group × subgroup variable) into linear regression models. Forest plots were created using Review Manager (RevMan) version 5.4 to visually summarize the subgroup analysis findings.

### Surgical technique

All surgeries were performed by the same senior surgical team at our center. Patients were placed in the prone position under local anesthesia (using 1% lidocaine).

### Visualized Reamer Group

**Figure 1:**
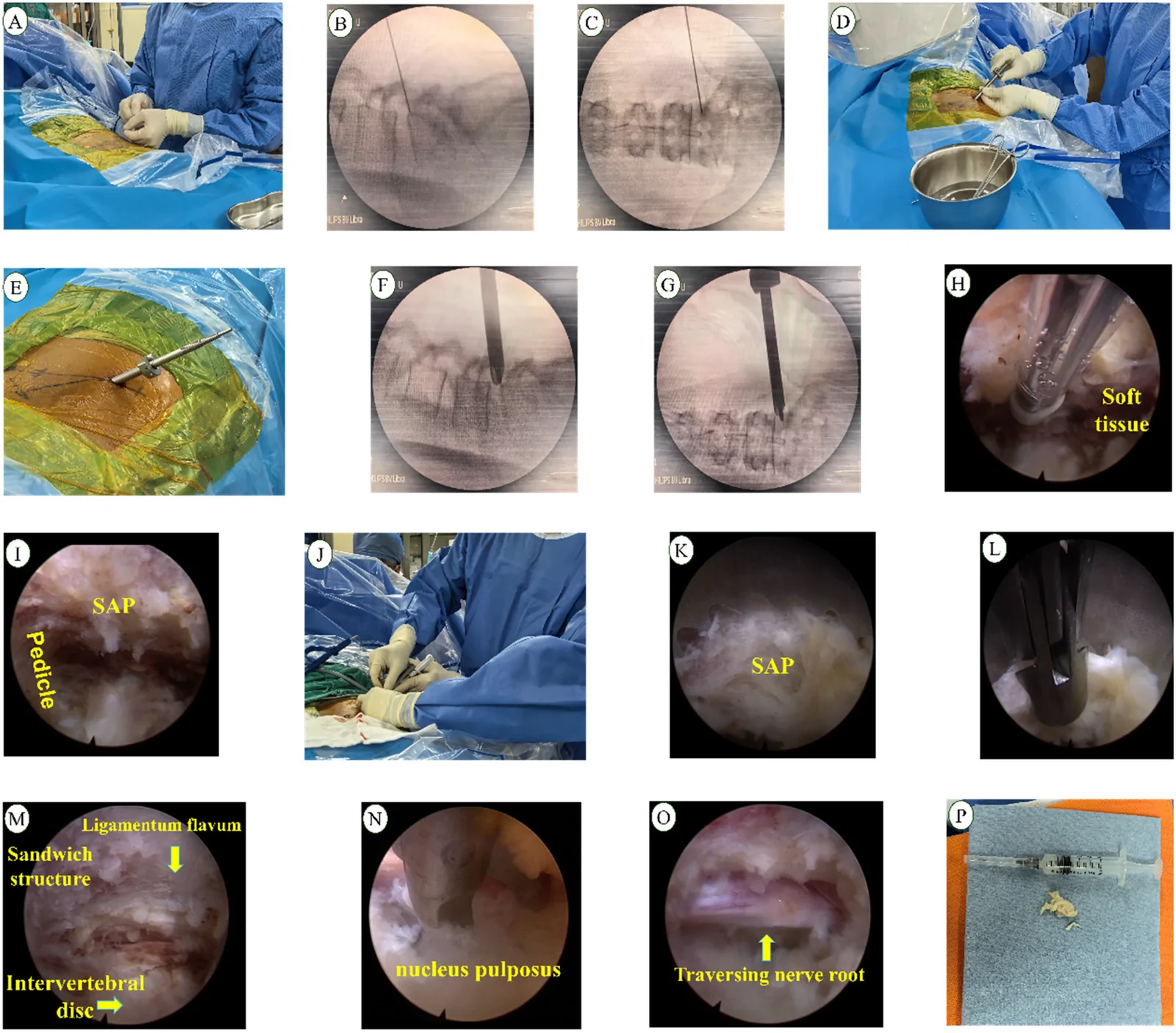
**(A)** puncture operation; **(B)** puncture lateral DR; **(C)** puncture anteroposterior DR; **(D)** cannulation operation; **(E)** appearance after cannulation; **(F)** cannulation lateral DR; **(G)** cannulation anteroposterior DR; **(H)** endoscopic debridement of soft tissue; **(I)** exposure of intervertebral foramen structures; **(J)** foraminoplasty operation; **(K)** bone resection; **(L)** removal of bone fragments with forceps; **(M)** sandwich structure after foraminoplasty; **(N)** removal of nucleus pulposus with forceps; **(O)** exposure of nerve root after decompression; and **(P)** excision of nucleus pulposus.

**Figure 2:**
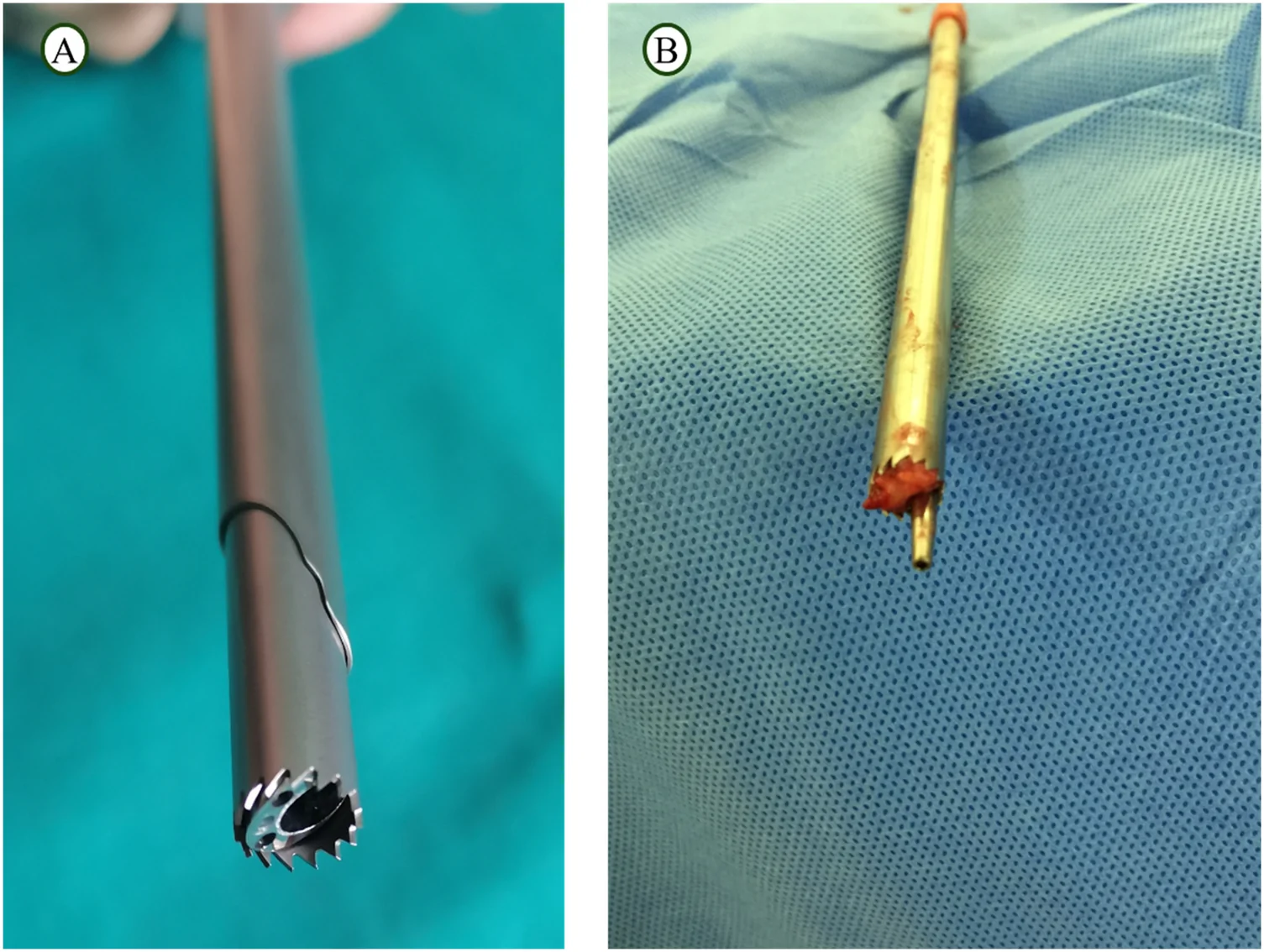
**(A)** Extra-endoscopic visualized reamer and **(B)** TESSYS reamer.

**Figure 3:**
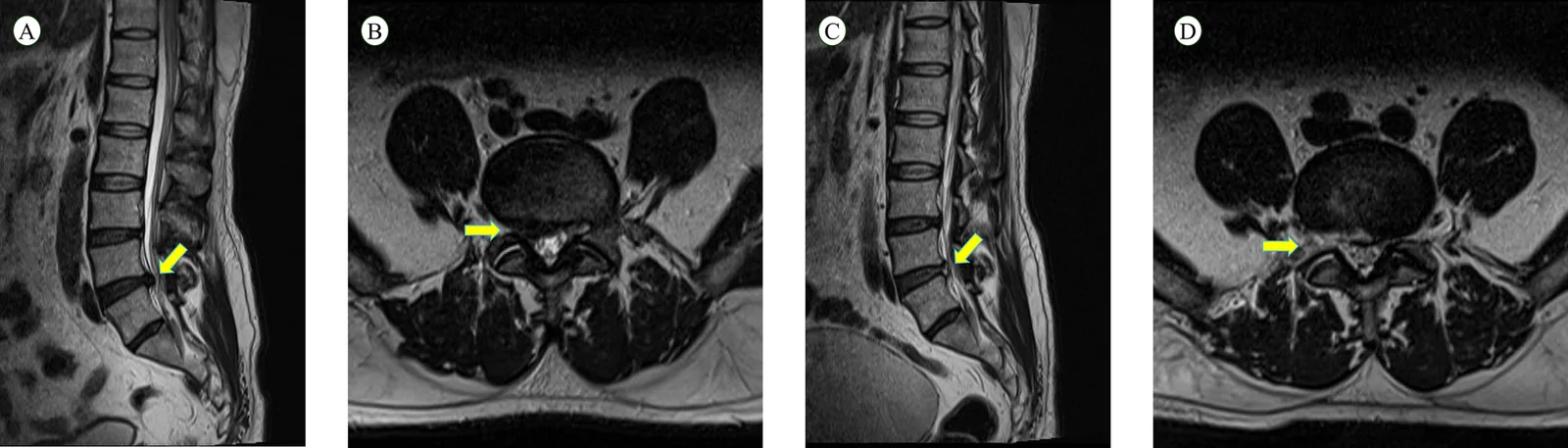
Visualized reamer group (right paracentral lumbar disc herniation): **(A)** preoperative lumbar MRI sagittal view; **(B)** preoperative lumbar MRI axial view; **(C)** postoperative lumbar MRI sagittal view; and **(D)** postoperative lumbar MRI axial view.

Following standard sterilization and draping, the target intervertebral foramen and puncture trajectory were identified and marked under C-arm fluoroscopy. The puncture point was situated approximately 10-12 cm lateral to the midline (Figure 1A). A puncture needle was advanced along the planned path at a 20°–40° angle relative to the coronal plane. Fluoroscopy confirmed the needle tip reached the lateral aspect of the superior articular process (SAP) of the lower vertebra at the target level (Figures 1B,C). After removing the stylet, a guidewire was inserted through the needle, which was then withdrawn. A series of sequential dilators were passed over the guidewire to establish a soft-tissue working channel (Figures 1D,E). After fluoroscopic confirmation of the dilator's position within Kambin's triangle (Figures 1F,G), the working cannula was inserted near the SAP. The endoscope and visualized reamer were introduced through the cannula (Figure 2A). Upon connecting and adjusting the light source and imaging system to obtain a clear view, soft tissues surrounding the foramen were carefully cleared under real-time endoscopic vision to expose the SAP (Figures 1H,I). The visualized reamer was then used to perform precise, on-demand bone resection from the SAP to enlarge the neuroforamen (Figures 1J–M). The working channel was advanced into the spinal canal. Discectomy and neural decompression were performed under continuous irrigation with 0.9% saline (Figure 1N), accompanied by radiofrequency coagulation for hemostasis. After confirming adequate decompression and free pulsation of the nerve root, the endoscope was partially withdrawn to probe the nerve root with a probe (Figures 1O,P). The operative field was irrigated, radiofrequency ablation was applied for annuloplasty, the working channel was removed, and the incision was sutured. Patients underwent an MRI examination before and after surgery (Figures 3A–D).

### TESSYS technique group

**Figure 4:**
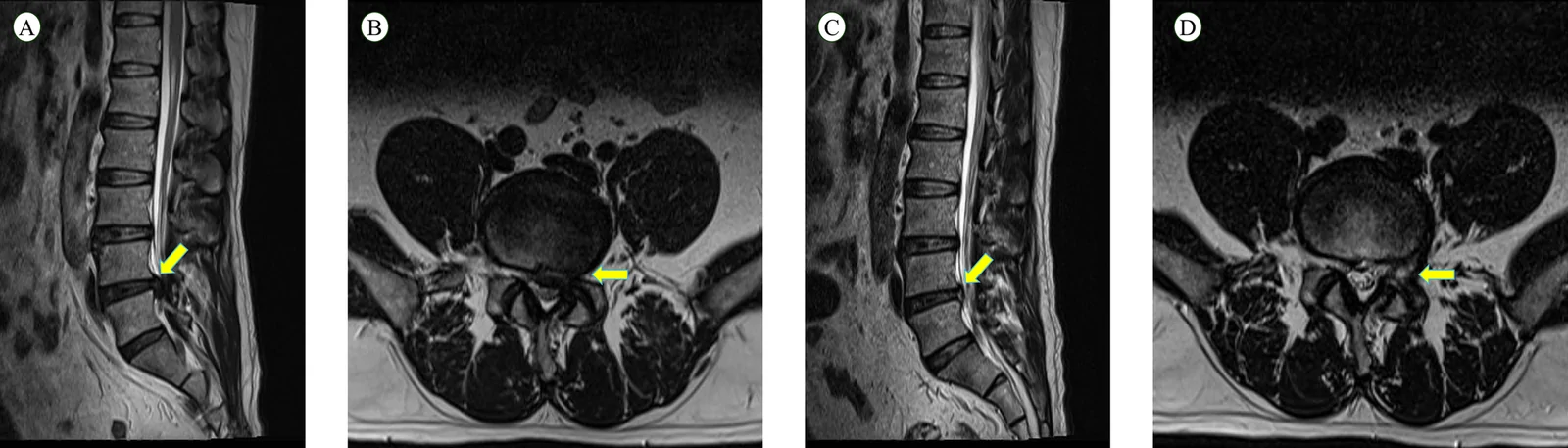
TESSYS technique group (left paracentral lumbar disc herniation); **(A)** preoperative lumbar MRI sagittal view; **(B)** preoperative lumbar MRI axial view; **(C)** postoperative lumbar MRI sagittal view; and **(D)** postoperative lumbar MRI axial view.

Preoperative preparation, patient positioning, anesthesia, initial puncture, and working channel establishment were identical to those in the visualized reamer group. The critical distinction lay in the foraminoplasty method: after puncture, a conventional reamer was used under repeated fluoroscopic guidance to perform the foraminoplasty, ensuring the reamer advanced toward the target without extending the tip beyond a line 5 mm medial to the medial border of the pedicle. After fluoroscopic confirmation of adequate foraminoplasty, the reamer was removed (Figure 2B), and the working channel was reinserted. Subsequent steps of discectomy, neural decompression, hemostasis, annuloplasty, and wound closure were identical to those in the visualized reamer group. Patients underwent an MRI examination before and after surgery (Figures 4A–D).

### Postoperative management

Patients were encouraged to ambulate with a lumbar brace on the first postoperative day to prevent thrombosis. Discharge typically occurred between postoperative days 2 and 5, at which time medications were prescribed: mecobalamin (to support nerve repair) and aescuven forte (to reduce edema and improve microcirculation). All patients were advised to avoid heavy lifting and strenuous activities for 3 to 6 months postoperatively.

## Results

A total of 140 patients were enrolled in this study (70 per group). No significant differences were observed in baseline characteristics between the two groups (Table 1). All surgeries were completed successfully, with a 12-month follow-up period for all participants.

**Table 1:** Comparison of baseline characteristics and perioperative outcomes between the Two groups.

Parameters	Visualized reamer group (*n* = 70)	TESSYS group (*n* = 70)	*P*-value	Statistical value
Age (years)	53.37 ± 15.62	50.54 ± 15.26	0.280	t = 1.084
Gender	—	—	0.726	*χ*^2^ = 0.122
Male	45	43	—	—
Female	25	27	—	—
Surgical segment	—	—	0.444	χ^2^ = 2.675
L1/2	0	0	—	—
L2/3	1	2	—	—
L3/4	5	7	—	—
L4/5	58	59	—	—
L5/S1	6	2	—	—
BMI (kg/m^2^)	25.19 ± 3.61	23.92 ± 3.98	0.051	t = 1.969
Operative time (min)	66.34 ± 7.65	76.06 ± 15.89	<0.001*	t = −4.610
Fluoroscopy times (times)	6.10 ± 0.90	12.06 ± 0.92	<0.001*	t = −38.764
Intraoperative blood loss (mL)	6.50 ± 2.71	6.84 ± 4.10	0.560	t = −0.584
Time from operation to discharge (days)	3.23 ± 2.09	2.87 ± 2.47	0.357	t = 0.924
Length of hospital stay (days)	5.74 ± 3.01	5.13 ± 3.00	0.228	t = 1.211
Post-op recurrence	1	2	0.559	χ^2^ = 0.341
Post-op lower limb dysesthesia	0	4	0.042*	χ^2^ = 4.118

VAS, visual analog scale; ODI, Oswestry disability index.

Data are presented as mean ± standard deviation.

* *P* < 0.05 vs. TESSYS group.

Compared to the TESSYS group, the visualized reamer group exhibited a significantly shorter mean operative time and required fewer intraoperative fluoroscopies (both *P* < 0.001). No significant differences were observed in intraoperative blood loss, time to discharge, or total hospital stay between the groups (Table 1).

Postoperative VAS scores for back and leg pain, as well as ODI scores, were significantly improved at all time points compared to preoperative baselines in both groups (*P* < 0.05). The visualized reamer group demonstrated superior improvement in leg VAS scores at 1 day and 1 month postoperatively relative to the TESSYS group. No significant differences were found between the groups at other time points for back VAS, leg VAS, or ODI scores (Table 2).

**Table 2:** Comparison of postoperative VAS, ODI scores, and modified MacNab criteria between the two groups.

Assessment/timepoint	Visualized reamer group (*n* = 70)	TESSYS group (*n* = 70)	*P*-value	Statistical value
Vas of back				
Pre-op	5.24 ± 3.15	5.81 ± 3.36	0.301	t = −1.038
1 day post-op	2.10 ± 1.46	2.49 ± 1.40	0.113	t = −1.597
1 month post-op	1.66 ± 1.36	1.96 ± 1.21	0.170	t = −1.379
3 months post-op	1.31 ± 1.36	1.59 ± 1.21	0.214	t = −1.249
6 months post-op	1.11 ± 1.40	1.26 ± 1.28	0.530	t = −0.630
12 months post-op	0.86 ± 1.49	0.93 ± 1.40	0.770	t = −0.293
VAS of leg				
Pre-op	7.14 ± 2.04	7.17 ± 2.05	0.934	t = −0.083
1 day post-op	2.37 ± 0.95	3.01 ± 1.06	<0.001*	t = −3.785
1 month post-op	2.14 ± 1.00	2.57 ± 1.20	0.023*	t = −2.300
3 months post-op	1.63 ± 1.13	1.94 ± 1.32	0.132	t = −1.514
6 months post-op	1.44 ± 1.22	1.56 ± 1.44	0.614	t = −0.506
12 months post-op	1.16 ± 1.51	1.51 ± 1.72	0.876	t = 0.156
ODI				
Pre-op	71.76 ± 18.20	67.89 ± 23.51	0.277	t = 1.091
1 day post-op	21.58 ± 8.55	22.80 ± 8.55	0.424	t = −0.802
1 month post-op	17.68 ± 9.14	19.35 ± 9.37	0.287	t = −1.069
3 months post-op	14.60 ± 10.17	15.88 ± 10.30	0.459	t = −0.742
6 months post-op	11.33 ± 11.48	12.43 ± 11.37	0.570	t = −0.569
12 months post-op	7.65 ± 13.02	8.25 ± 13.75	0.793	t = −0.263
Modified MacNab criteria				
Excellent	57	56	—	—
Good	7	3	—	—
Fair	5	8	—	—
Poor	1	3	—	—
Excellent/good rate	91.43%	84.29%	0.196	χ^2^ = 1.674

post-op, postoperative.

Value are presented as mean ± standard deviation.

* *P* < 0.05 vs. TESSYS group.

Based on the modified MacNab criteria, outcomes in the visualized reamer group were rated as excellent in 57 cases (81.43%), good in 7 (10.00%), fair in 5 (7.14%), and poor in 1 (1.43%). Correspondingly, in the TESSYS group, outcomes were excellent in 56 cases (80.00%), good in 3 (4.29%), fair in 8 (11.43%), and poor in 3 (4.28%). The excellent-good rate did not differ significantly between the groups (*P* > 0.05, Table 2).

Regarding complications, one case of ipsilateral recurrence in the visualized reamer group was managed conservatively with symptom resolution. In contrast, the TESSYS group had two recurrence cases, both of which were managed conservatively. Additionally, four cases in the TESSYS group experienced postoperative lower limb dysesthesia, all of which resolved with symptomatic treatment. No serious complications such as dural tears, wound hematomas, or surgical site infections occurred in either group (Table 1).

**Figure 5:**
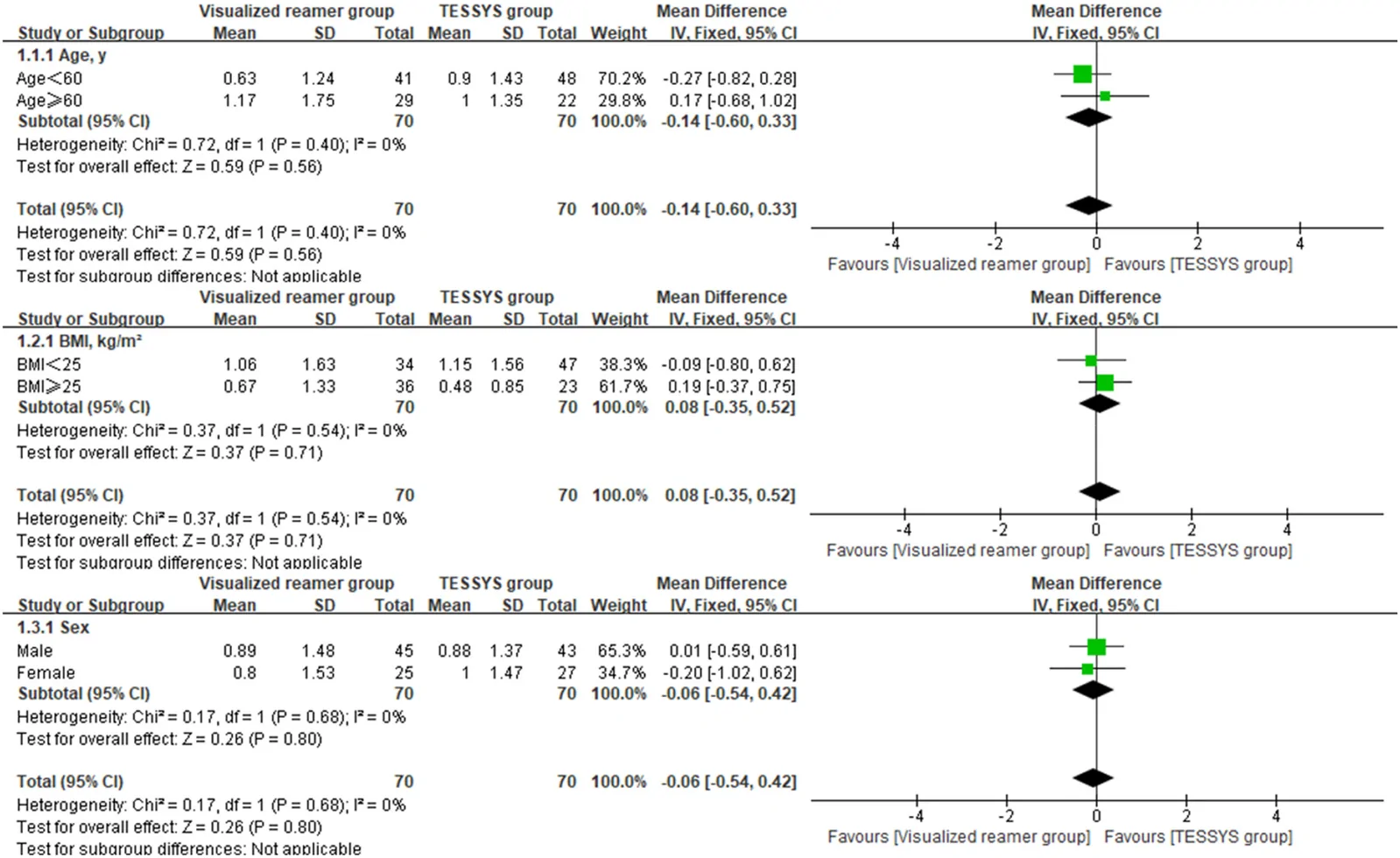
Forest plot of subgroup analysis for back VAS scores at 12 months postoperatively.

**Figure 6:**
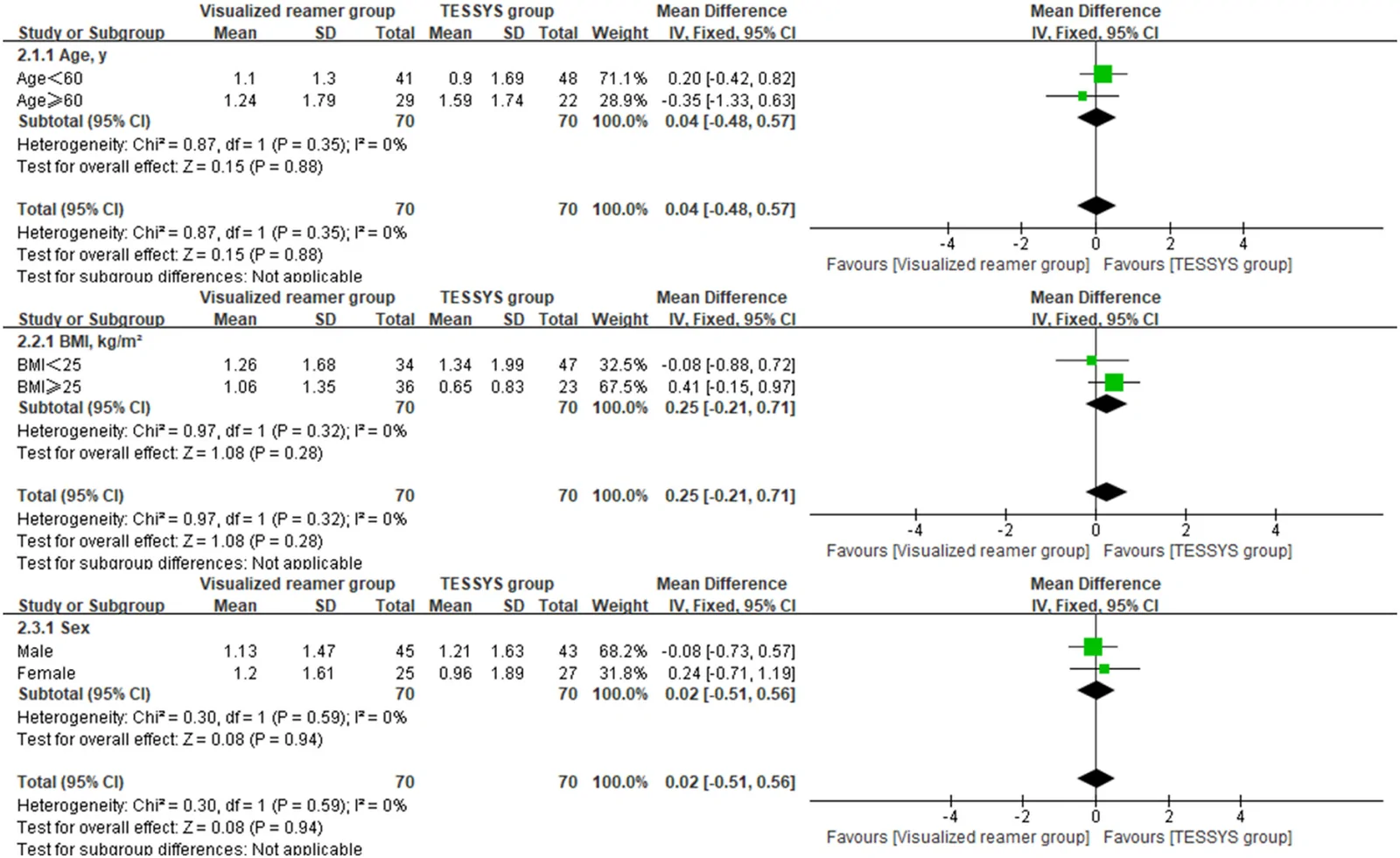
Forest plot of subgroup analysis for leg VAS scores at 12 months postoperatively.

**Figure 7:**
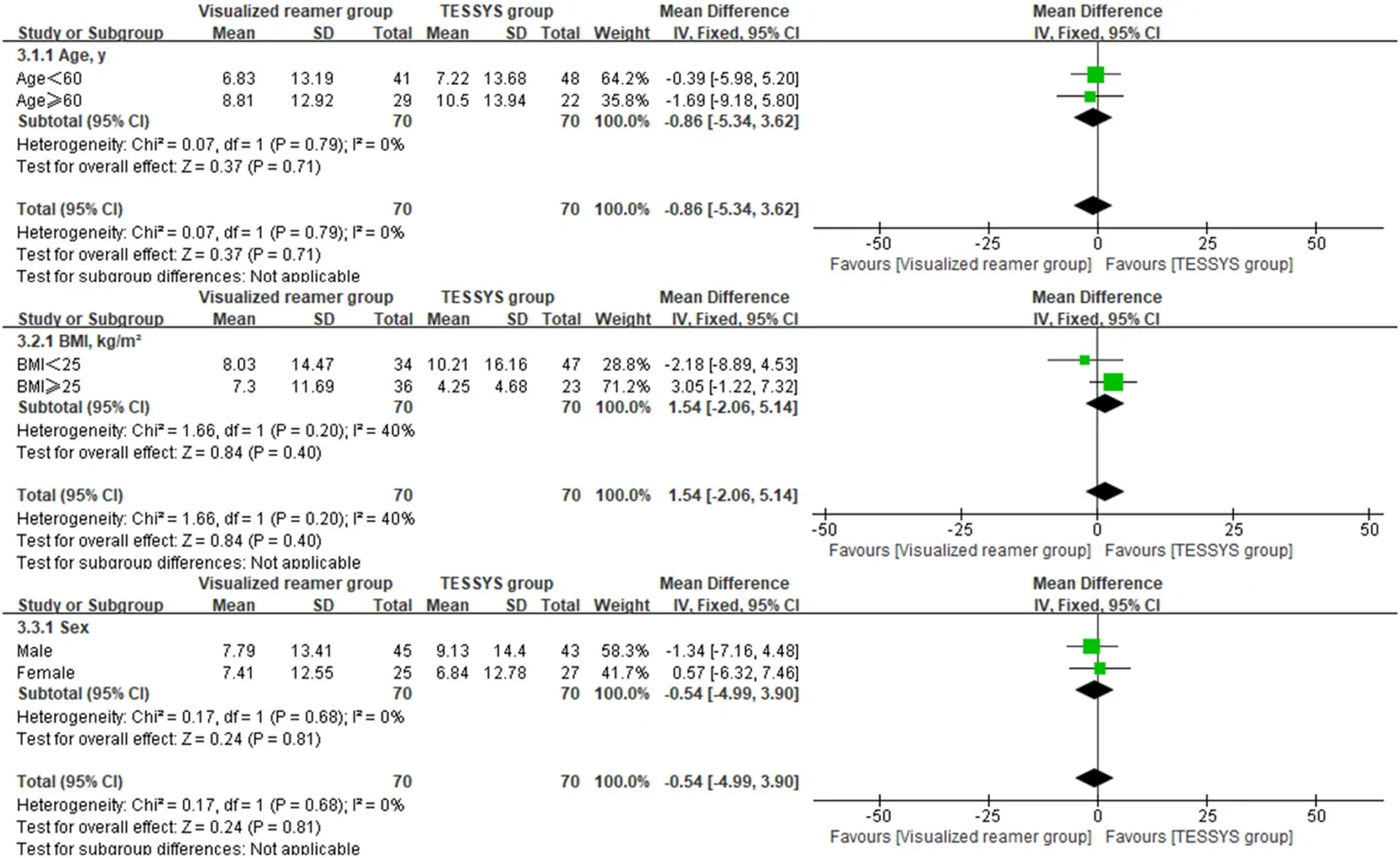
Forest plot of subgroup analysis for ODI at 12 months postoperatively.

**Figure 8:**
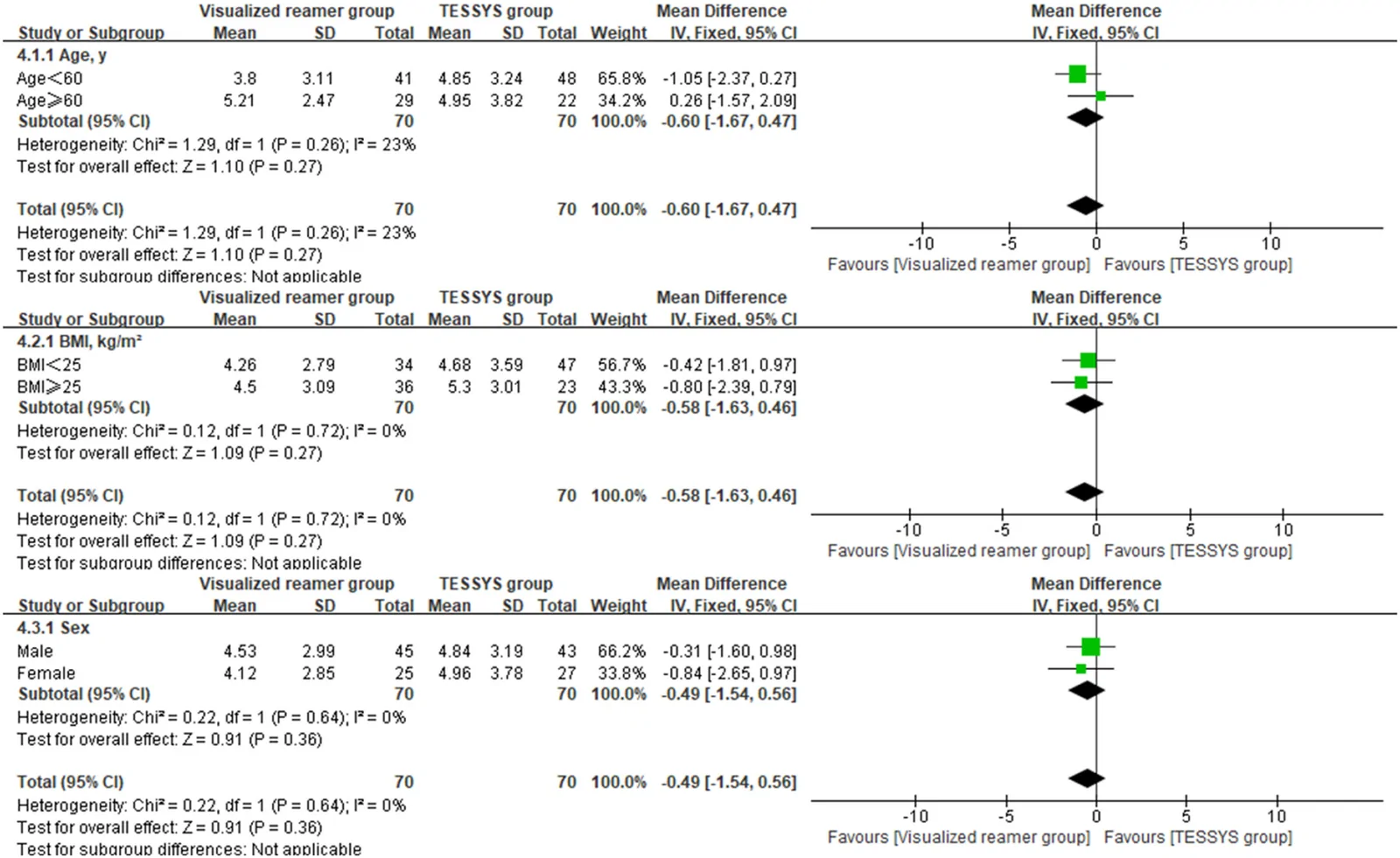
Forest plot of subgroup analysis for change in back VAS scores (Δ).

**Figure 9:**
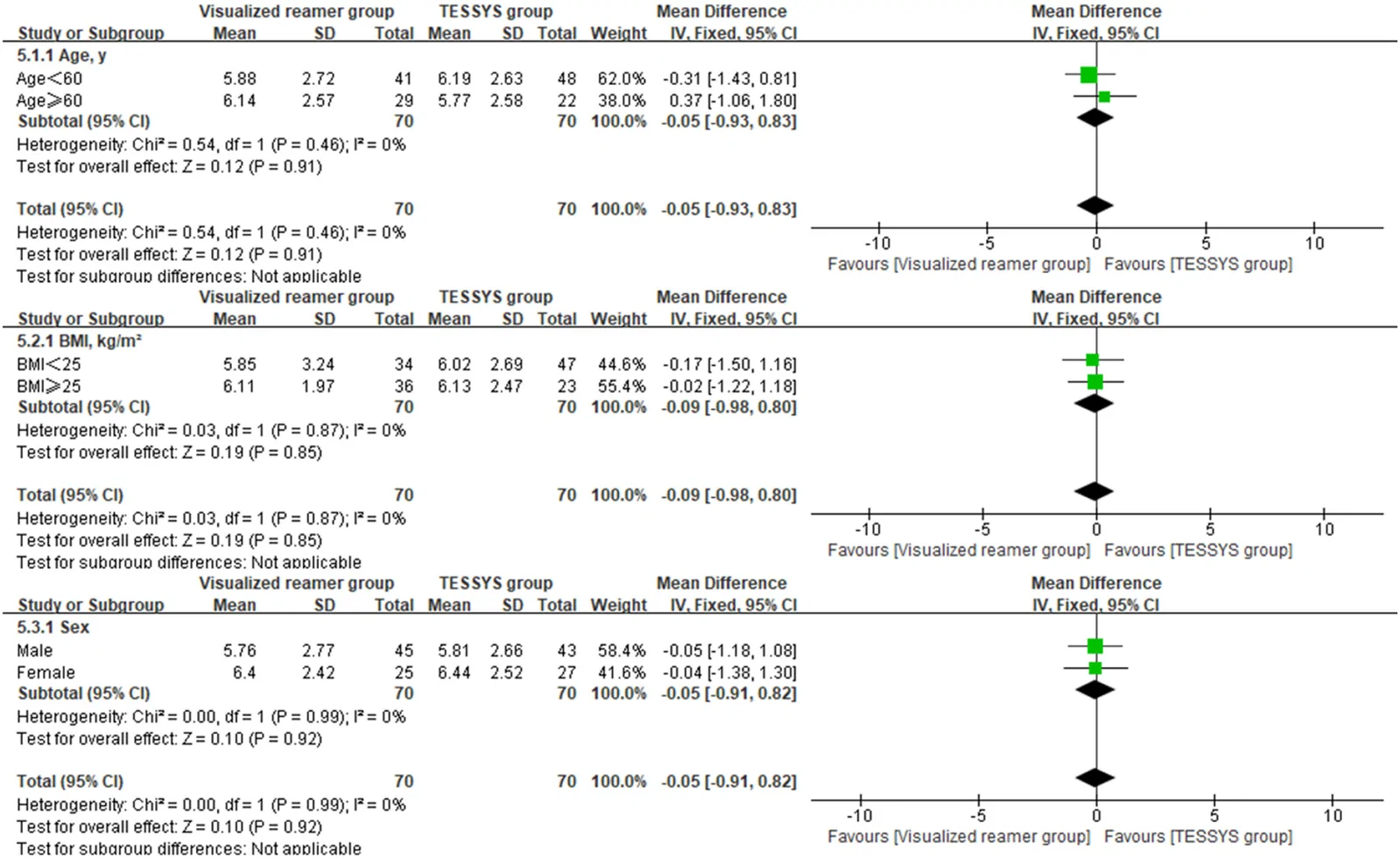
Forest plot of subgroup analysis for change in leg VAS scores (Δ).

**Figure 10:**
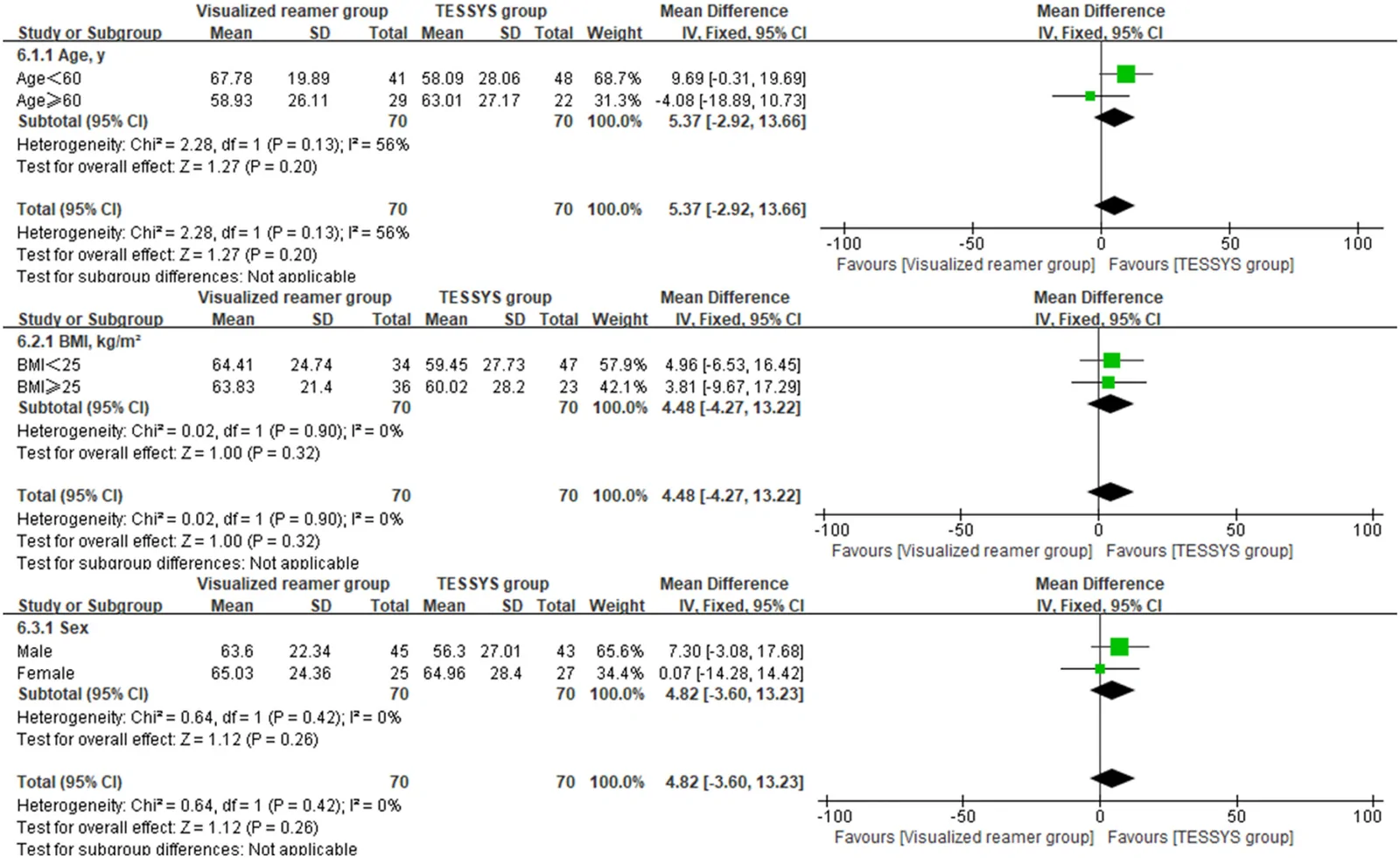
Forest plot of subgroup analysis for change in ODI (Δ).

Interaction analysis indicated no significant interaction effects between the treatment method and any subgroup variable (age, BMI, sex) for any primary outcome at 12 months (*P* > 0.05 for all interactions, Table 3), suggesting that the benefits of the visualized reamer technique in terms of surgical efficiency and early leg pain relief were consistent across all patient subgroups. Forest plots visually supported this finding. As shown in Figure 5 for back VAS scores at 12 months, the confidence intervals for the difference between groups widely overlapped and crossed the line of no effect across all subgroups (age ≤ 60 or >60, BMI < 25 or ≥25 kg/m^2^, male or female). Similar patterns of treatment effect consistency were observed for all other outcome measures, no significant differences between subgroups (Figures 6–10).

**Table 3:** Tests for interaction in prespecified subgroups.

Subgroup variable	Outcome measure	*P-*value for interaction
Age	VAS of back at 12 months post-op	0.396
	VAS of leg at 12 months post-op	0.336
	ODI at 12 months post-op	0.782
	Δ VAS of back	0.246
	Δ VAS of leg	0.471
	Δ ODI	0.127
BMI	VAS of back at 12 months post-op	0.578
	VAS of leg at 12 months post-op	0.397
	ODI at 12 months post-op	0.264
	Δ VAS of back	0.728
	Δ VAS of leg	0.872
	Δ ODI	0.898
Sex	VAS of back at 12 months post-op	0.687
	VAS of leg at 12 months post-op	0.584
	ODI at 12 months post-op	0.684
	Δ VAS of back	0.630
	Δ VAS of leg	0.988
	Δ ODI	0.417

## Discussion

This study compared the efficacy and safety of visualized reamer foraminoplasty with the conventional TESSYS technique in TELD for LDH. The findings indicated that both techniques resulted in significant clinical improvement throughout the 1-year follow-up. However, the visualized reamer technique demonstrated superior surgical efficiency and early postoperative recovery. Specifically, it was associated with a shorter operative time (66.34 ± 7.65 min vs. 76.06 ± 15.89 min, *P* < 0.05) and fewer instances of intraoperative fluoroscopy (6.10 ± 0.90 vs. 12.06 ± 0.92, *P* < 0.05) relative to the TESSYS technique. Moreover, improvement in leg pain VAS scores was more pronounced in the visualized reamer group at 1 day and 1 month postoperatively (*P* < 0.05), suggesting enhanced early pain relief. Prespecified subgroup analyses based on age, BMI, and sex showed no significant interaction effects for any primary outcome at 12 months (all *P*-interaction > 0.05) indicating that the benefits of the visualized reamer technique were consistent across different patient demographics. These results support the generalizability of this technique in broader clinical practice. In summary, visualized reamer foraminoplasty represents a refinement of TELD, improving surgical efficiency and early recovery while maintaining consistent outcomes across diverse patient populations.

The reliance on repeated fluoroscopy during foraminoplasty represented a limitation of the TESSYS technique. While the radiation exposure from a single procedure poses a low risk to any individual patient, the long-term cumulative occupational exposure for the surgical team, who routinely perform these procedures, represents a defined health concern. Evidence suggests that such cumulative exposure is associated with an increased risk of long-term adverse effects, including malignancies and genetic alterations (14–16). Consequently, reducing intraoperative fluoroscopy is of critical importance for protecting the surgical team from occupational radiation hazards.

In contrast, fluoroscopy in the visualized reamer technique was predominantly required only for the initial puncture and instrument positioning. The critical foraminoplasty step is subsequently performed under direct endoscopic visualization. This shift in technique enabled surgeons to clearly identify key anatomical landmarks, such as the ventral surface of the superior articular process and the superior margin of the pedicle, thereby facilitating precise, targeted bone resection under a clear visual field. This technological advancement translated into significant reductions in radiation exposure. Previous clinical studies have reported an average number of fluoroscopic shots per procedure for TESSYS ranging from 18.7 to 33.98 (17, 18). In comparison, our study confirmed that the visualized reamer technique significantly reduced this requirement, also resulting in a shorter operative time (*P* < 0.05). Furthermore, the greater variability in operative time (standard deviation: 15.89 vs. 7.65 min) observed in the TESSYS group suggests lower procedural predictability, likely reflecting its reliance on fluoroscopic guidance. In contrast, continuous direct visualization with the reamer appears to facilitate more consistent and controllable procedure times, thereby enhancing overall operative efficiency. This finding is consistent with another study by Lin et al. utilizing a full-endoscopic visualized reamer for foraminoplasty in elderly patients with foraminal stenosis, which reported an average of 3.21 fluoroscopies per procedure (19). Similarly, Shi et al. (17) reported averages of only 4.9 fluoroscopies and an operative time of 78.0 min with a visualized reamer, which were lower than TESSYS values. Yang et al. (20) also demonstrated that the visualized reamer technique resulted in lower radiation exposure and operative time compared to a progressive bone drilling system. Chang et al. compared the efficacy of extra-endoscopic trephine (EET) and endoscopic drill (ED) techniques in full-endoscopic foraminoplasty for single-segment lumbar disc herniation, and found that while both techniques yielded comparable clinical outcomes, the EET technique was associated with shorter operative and foraminoplasty times (21). A recent meta-analysis further confirmed that visualized foraminoplasty significantly reduced both operative time and fluoroscopy compared to the TESSYS technique (22). The consistency of these findings across studies underscored that the visualized reamer technique fundamentally enhanced surgical efficiency and safety by transforming foraminoplasty from a “fluoroscopy-dependent” to a “visually-guided” procedure.

The TESSYS technique, as a classic transforaminal approach, has demonstrated well-established efficacy through long-term clinical application (23–25). In our study, both groups exhibited significant postoperative improvement in all outcome measures compared to preoperative baselines (*P* < 0.05). The superior improvement in leg VAS scores observed in the visualized reamer group at 1 day and 1 month postoperatively suggests better early pain relief. This advantage may have been attributed to the greater precision of decompression and foraminoplasty afforded by direct visualization.

The key innovation of the visualized reamer technique was its capacity to enable precise operative control under direct endoscopic vision. Surgeons can first manage peri-foraminal soft tissues (e.g., ligaments, vasculature) under high-definition visualization, thereby enlarging the working space while reducing risks to neurovascular structures and establishing a safer, clearer environment for subsequent decompression. Following precise bone resection, the endoscope provides a clear view of a characteristic three-layered anatomical configuration (sandwich structure): the ligamentum flavum superiorly, the nerve root and accompanying vessels in the middle, and the disc tissue inferiorly. For surgeons, clear identification of this anatomy serves as an important intraoperative landmark, confirming adequate decompression and providing a reliable guide for subsequent discectomy and nerve root exploration.

Furthermore, the dorsal root ganglion (DRG), located within the intervertebral foramen, is highly sensitive to mechanical compression and irritation, serving as a key basis for radicular pain and dysesthesia (26, 27). The visualized reamer technique, by providing real-time endoscopic visualization, enables surgeons to identify and proactively avoid these sensitive structures during foraminoplasty, thereby minimizing blind mechanical stimulation to the DRG. This reduction in intraoperative neural irritation likely represents an additional important mechanism contributing to the more rapid relief of early postoperative leg pain observed in the visualized reamer group. The Mid-term functional and pain scores ultimately converged between the two groups (*P* > 0.05), indicating comparable Mid-term efficacy, the visualized reamer technique provided the distinct benefit of reducing early postoperative pain, offering a clinical advantage during the initial recovery phase (28). This observation was supported by several clinical studies (17, 28–32) which have also reported that TELD with visualized reamer foraminoplasty effectively alleviated pain and promoted functional recovery.

The key to achieving precise foramination with the visualized reamer technique lies in accurately placing the puncture needle lateral to the superior articular process of the target segment and advancing the working channel into Kambin's triangle following precise initial fluoroscopic localization. Subsequently, insertion of the visualized reamer and endoscope allows for direct identification of anatomical landmarks and performance of foraminoplasty under visual guidance. The efficacy of this technique relies on meticulous preoperative imaging planning and real-time intraoperative anatomical recognition, and also necessitates that the surgeon possesses proficient puncture and cannulation skills. In contrast, the conventional TESSYS technique, as an established method of fluoroscopically-guided targeted foraminoplasty, similarly requires high-quality imaging for initial localization. Its process involves continuous fluoroscopy during foraminoplasty to confirm the position of the reamer tip relative to the pedicle and midline, thereby ensuring advancement toward the target while remaining within safe boundaries. Therefore, the two techniques embody different pathways to precision: the visualized reamer technique emphasizes the combination of accurate initial localization with real-time visual control of anatomy, whereas the TESSYS technique depends on continuous intraoperative fluoroscopic feedback and trajectory adjustment. Both approaches require standardized execution to achieve effective and safe foraminoplasty.

We also analyzed postoperative complications, with a specific focus on recurrence and postoperative lower limb dysesthesia. Regarding recurrence, while the objective of TELD is to remove herniated disc material to achieve neural decompression, patients with LDH remain susceptible to recurrence due to factors such as age-related degeneration, inadequate postoperative rest, and obesity (33, 34). In our cohort, recurrence occurred in one patient in the visualized reamer group compared to two in the TESSYS group (1.43% vs. 2.86%, *P* > 0.05). Given the limited number of events and the multifactorial nature of recurrence, no definitive association could be established between the foraminoplasty technique and recurrence risk based on these data alone. Postoperative lower limb dysesthesia is another known complication of TELD (35), potentially related to nerve root traction or direct stimulation during surgery (10, 36). Silav et al. (37) reported that postoperative dysesthesia often results from instrument stimulation and improper manipulation. In our study, no cases of lower limb dysesthesia were observed in the visualized reamer group, whereas four cases (5.71%) occurred in the TESSYS group (*P* < 0.05). This finding is consistent with previous reports: Fan et al. (34) noted a complication rate of approximately 9.76% for the TESSYS technique, with persistent low back pain and lower limb dysesthesia occurring in 3.79% of cases. Chen et al. (38) observed no neural complications in 25 patients treated with a visualized trephine foraminoplasty technique for migrated fragments or foraminal stenosis. Similarly, Ouyang et al. (30) reported no complications in the visualized reamer group in a direct comparative study. The ability to clearly identify the nerve root and adjacent structures under endoscopic vision may help avoid inadvertent neural irritation, suggesting that the visualized reamer technique offers a safer surgical alternative. It should be noted that this study has a retrospective design and the absolute number of complication events was low. Specifically, a *post-hoc* power analysis for the between-group difference in postoperative lower limb dysesthesia incidence revealed limited statistical power (Power=37%). Therefore, the relationship between the foraminoplasty method and complication rates, as well as the potential safety advantage suggested here, warrant further validation through larger clinical studies and biomechanical investigations.

Most comparative studies on foraminoplasty techniques have primarily focused on outcomes in the overall patient population (20, 30, 39–41), often omitting subgroup analyses based on key demographic characteristics, which limited the exploration of potential heterogeneity in treatment effects. This study incorporated a prespecified subgroup analysis to evaluate whether the benefits of the visualized reamer technique varied according to baseline demographics, including age, BMI, and sex. The results demonstrated that none of the interaction terms reached statistical significance (*P* > 0.05 for all), indicating consistent treatment effects across these subgroups. According to the criteria proposed by Sun et al. (42), this finding of no significant interaction was considered credible and clinically relevant, as the analysis was prespecified and involved a limited number of clinically rational subgroup variables, thereby reducing the risk of false-positive results from multiple testing.

This consistency of effect is supported by existing research. A meta-analysis by Feng et al. (43) showed no significant difference in VAS and ODI improvements between obese and non-obese patients following TELD, supporting the efficacy and safety of the procedure across BMI categories. Xu et al. (44) observed that while younger LDH patients experienced greater pain relief shortly after TELD compared to older patients, the influence of age diminished with longer follow-up, highlighting the durable benefit of the technique irrespective of age. Furthermore, Kapetanakis et al. (45) found that both male and female patients showed significant improvements in quality of life after TELD, with no notable sex-based differences.

From a clinical perspective, the principal advantage of the visualized reamer technique is its visual operative paradigm, which reduces the uncertainty associated with fluoroscopy-dependent methods and provides a safer, more precise surgical environment. This fundamental benefit is inherently universal, which may explain the consistent treatment effects observed across diverse patient subgroups. Although specific biological mechanisms explaining the lack of effect modification by age, BMI, or sex are currently unavailable, the accumulated evidence suggests that surgeons can consider the visualized reamer a reliable and broadly applicable technique, rather than one limited to specific patient subgroups.

## Limitations

This study has several limitations. Its single-center retrospective nature may introduce selection bias. The sample size, though adequate for primary analyses, may lack power for detecting subtle subgroup interactions. Additionally, the retrospective sequential application of the two techniques means that the comparison could be influenced by temporal factors, such as the surgical team's growing familiarity and efficiency with the overall TELD workflow over time. Finally, the 1-year follow-up is sufficient for mid-term outcomes but inadequate for assessing long-term complications or recurrence.

## Conclusion

Both surgical techniques were effective for LDH. However, the visualized reamer technique offered superior efficiency—reducing operative time and radiation exposure—and better early pain control, with comparable mid-term outcomes to TESSYS. These benefits were consistent across patient subgroups, establishing it as an advanced refinement of the established TESSYS approach.

## Data Availability

The raw data supporting the conclusions of this article will be made available by the authors, without undue reservation.
