# Correction: Efficacy of visualized reamer foraminoplasty in transforaminal endoscopic lumbar discectomy: a retrospective controlled study

**DOI:** 10.3389/fsurg.2026.2005504

**Published:** 2026-10-26

**Authors:** Honglin Liu, Yuxiang Hu, Yang Zhan, Zhixin Kang, Zhuoxuan Zhang, Chengyu Huang, Ningjing Zeng, Yang Xiao, Juntao Ma, Zibo Gao, Hao Liu, Guoyi Su, Yongpeng Lin, Zhirong Fan, Dingkun Lin, Yihao Liang, Yongjin Li

**Affiliations:** 1 Second Clinical Medical College, Guangzhou University of Traditional Chinese Medicine, Guangzhou, China; 2 Gansu University of Chinese Medicine, Lanzhou, China; 3 The Second Affiliated Hospital of Guangzhou University of Chinese Medicine, Guangzhou, China; 4 Ruikang Hospital Affiliated to Guangxi University of Chinese Medicine, Nanning, China

**Keywords:** foraminoplasty, Lumbar disc herniation, minimally invasive spine surgery, transforaminal endoscopic lumbar discectomy, visualized reamer

There were mistakes in Table 1 as published. In the “Surgical segment” row, the cells for both groups were incorrectly given as “0” instead of “–” (dash), indicating non-applicable entries. Similarly, in the “Gender” row, the summary cells should also be displayed as “–”. Finally, for the TESSYS group, the number of cases at L2/3 was given as “0” instead of the correct value of “2”.

The corrected Table 1 appears below.

**Table 1:** Comparison of baseline characteristics and perioperative outcomes between the Two groups.

Parameters	Visualized reamer group (*n* = 70)	TESSYS group (*n* = 70)	*P*-value	Statistical value
Age (years)	53.37 ± 15.62	50.54 ± 15.26	0.280	t = 1.084
Gender	—	—	0.726	*χ*² = 0.122
Male	45	43	—	—
Female	25	27	—	—
Surgical segment	—	—	0.444	χ² = 2.675
L1/2	0	0	—	—
L2/3	1	2	—	—
L3/4	5	7	—	—
L4/5	58	59	—	—
L5/S1	6	2	—	—
BMI (kg/m²)	25.19 ± 3.61	23.92 ± 3.98	0.051	t = 1.969
Operative time (min)	66.34 ± 7.65	76.06 ± 15.89	<0.001*	t = −4.610
Fluoroscopy times (times)	6.10 ± 0.90	12.06 ± 0.92	<0.001*	t = −38.764
Intraoperative blood loss (mL)	6.50 ± 2.71	6.84 ± 4.10	0.560	t = −0.584
Time from operation to discharge (d)	3.23 ± 2.09	2.87 ± 2.47	0.357	t = 0.924
Length of hospital stay (d)	5.74 ± 3.01	5.13 ± 3.00	0.228	t = 1.211
Post-op recurrence	1	2	0.559	χ² = 0.341
Post-op lower limb dysesthesia	0	4	0.042*	χ² = 4.118

VAS, visual analog scale; ODI, Oswestry disability index.

Data are presented as mean ± standard deviation.

* *P* < 0.05 vs. TESSYS group.

The original version of this article has been updated.

